# A Case of Submandibular Leiomyosarcoma, Mimicking an Abscess, in a Ball Python (*Python regius*)

**DOI:** 10.3390/vetsci8100224

**Published:** 2021-10-11

**Authors:** Jun Kwon, Sang Wha Kim, Sang Guen Kim, Hyoun Joong Kim, Sung Bin Lee, Jeong Woo Kang, Won Joon Jung, Sib Sankar Giri, Kyunglee Lee, Se Chang Park

**Affiliations:** 1 Laboratory of Aquatic Biomedicine, Research Institute for Veterinary Science, College of Veterinary Medicine, Seoul National University, Seoul 08826, Korea; kjun1002@snu.ac.kr (J.K.); kasey.kim90@gmail.com (S.W.K.); imagine5180@gmail.com (S.G.K.); lsbin1129@naver.com (S.B.L.); kck90victory@naver.com (J.W.K.); cwj0125@gmail.com (W.J.J.); giribiotek@gmail.com (S.S.G.); 2 Department of Marine Life Science, Jeju National University, Jeju 63243, Korea; hjoong1@nate.com; 3 Cetacean Research Institute (CRI), National Institute of Fisheries Science (NIFS), Ulsan 44780, Korea; dorijjang19@gmail.com

**Keywords:** submandibular leiomyosarcoma, abscess, *Python regius*, immunohistochemistry

## Abstract

A two-year-old ball python with a submandibular mass was evaluated. Fine needle aspiration resulted in debris containing purulent materials and bacterial cells on cytology. Radiography demonstrated multi-focal radiopaque lesions in the mass, which were suspected to be mineralization; there was an absence of mandibular invasion or lung involvement. Gross examination of the surgically excised mass revealed a multi-nodular, well-circumscribed lesion with purulent material. The postoperative recovery was uneventful. The histopathological examination followed by immunohistochemistry analysis gave a diagnosis of leiomyosarcoma. As tumors containing purulent materials can be confused with an abscess, diagnostic confirmation with various diagnostical tools should be considered.

## 1. Introduction

Ball python (*Python regius*), a terrestrial reptile species, is native to western and central Africa [1]. This species is one of the most popular reptile pets worldwide because of its docility and relative ease of care [1,2]. As the population and, consequently, trade of this species increased, there has been a considerable rise in its medical and zoological studies. Although exotic animal medicine has recently advanced, neoplasms in reptilian populations continue to be a big challenge. Insufficient knowledge of reptile oncology makes clinicians adapt treatment protocols from companion animal oncology practices [3,4]. Therefore, reference studies with companion animals and human oncology should be performed for effective treatment.

Leiomyosarcoma is a malignant tumor originating in the smooth muscle. In human medicine, it is a common type of soft tissue sarcoma, accounting for approximately 10% of sarcoma cases [5]. Because it originates in the smooth muscle, reports have mainly described leiomyosarcomas of the alimentary track, liver, spleen, or other organs containing smooth muscle [6]. Subcutaneous leiomyosarcomas are rare, accounting for approximately 3% of all soft tissue sarcomas in humans [7]. Although there is a paucity of information on the exact location or age-related propensities of subcutaneous leiomyosarcomas, many studies have demonstrated a predilection for age between 40–60 years and a likelihood of it being found in the thighs or extremities [8,9]. Development of leiomyosarcoma in the face is exceedingly rare [8].

In this study, we describe a case of putative submandibular subcutaneous leiomyosarcoma in a 2-year old ball python and present its clinical, histopathological, and radiographic features.

## 2. Case Presentation

**Figure 1:**
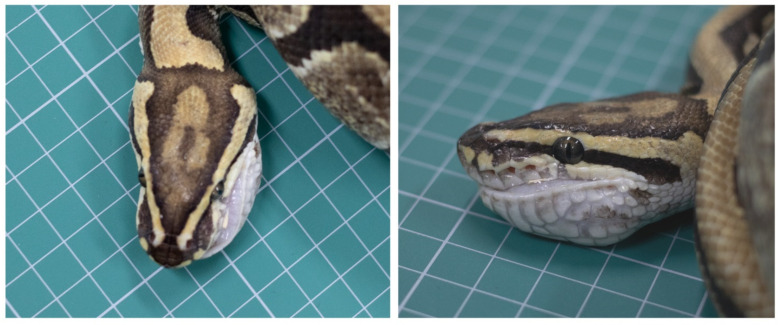
Clinical examination of the snake revealed a 2 × 3.5 cm-sized mass in the left submandibular region.

A 2-year-old, 101-cm long, ball python (*Python regius*), weighing 1.2 kg, was evaluated for a submandibular mass (Figure 1). The mass measured 2 × 3.5 cm in size and was located in the left submandibular region; on clinical examination, it was hard on palpation and multi-lobular. Clinical history suggested that the size of the mass had not changed since the owner purchased it one year ago. The patient had no other clinical symptoms, such as loss of appetite or any eating disorder, and was active, aggressive, and responsive.

**Figure 2:**
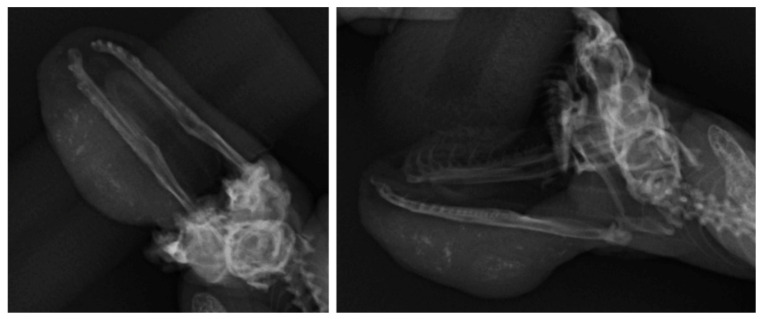
Radiographic examination of the submandibular mass revealed the presence of multi-focal mineralization in the area of the lesion. No evidence of mandibular invasion and lung metastasis was observed.

Radiographs were performed to evaluate the submandibular mass and lungs. The submandibular mass showed multi-focal radiopaque lesions, which were suspected to be calcifications, and there was no evidence of mandibular bone invasion or lung metastasis (Figure 2). The patient was anesthetized via chamber induction with isoflurane. Fine needle aspiration (FNA) was performed for the cytological study. However, only tissue debris and purulent materials, such as degenerated heterophiles and bacteria, were seen on cytology. Bacterial cultures were performed on Columbia agar containing 5% sheep blood under both aerobic and anaerobic conditions at 27 °C for 24 h. One colony of the bacteria was obtained by colony morphology. The bacteria were identified as *Klebsiella oxytoca* by 16s rRNA gene sequencing (Macrogen, Seoul, South Korea).

**Figure 3:**
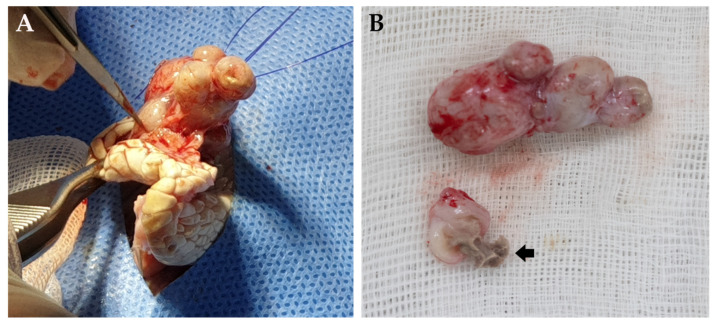
Gross examination of the mass after surgical removal. (**A**) Multinodular appearance of the mass. (**B**) Pus visible (arrow).

Surgical excision of the mass was performed after 3 days of fasting. Anesthesia was induced by chamber induction and maintained with isoflurane and oxygen during the procedure. After making the incision, a multi-nodular mass was observed in the subcutaneous region without any infiltration or metastasis into the surrounding tissue and was well-delineated. The mass was surgically removed (Figure 3). Cephalexin (20 mg/kg; PO, q12h) and meloxicam (0.2 mg/kg; PO, q24h) were administered for 7 days. The patient was hospitalized for 5 days after surgery. There was no evidence of complications, and the patient’s appetite was normal.

Macroscopically, the mass was whitish in color, well-demarcated, and consisted of multiple nodules. The nodule sizes were different, but they were strongly adhered together. Additionally, the nodules on cut sections were found to be capsular structures containing caseous abscesses that were occupying approximately 30% of the mass. The resected mass was examined microbiologically and histologically. Material from the abscesses was cultured, and the isolates were also identified as *K. oxytoca*. 

**Figure 4:**
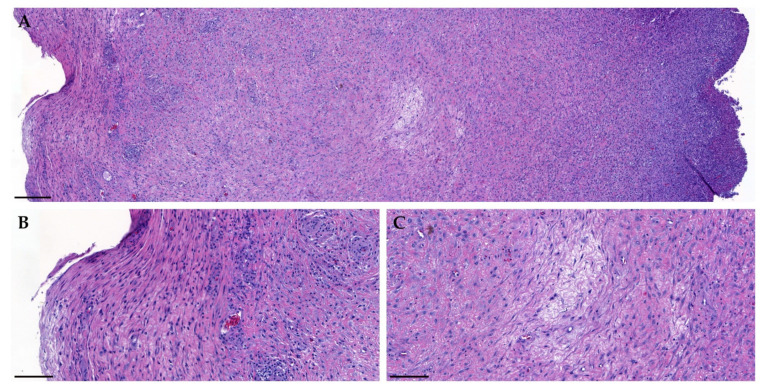
Histological examination of the mass (hematoxylin and eosin stain). (**A**) Low power (×7) of the mass. Inner border (right side) of the mass demonstrates massive heterophile infiltration (scale bar = 200 μm). (**B,C**) High power (×30) of the mass. The neoplasm was poorly demarcated, moderately to densely cellular, infiltrative, and composed of neoplastic spindle cells that formed loose interlacing streams and bundles within a mild fibrovascular stroma (scale bar = 100 μm).

Histological examination of the resected mass was performed by the Korea Vet lab (KVL, Seongnam, South Korea) (Figure 4). H & E stained sections revealed a connective tissue stroma with a capsular structure. The tumor was an unencapsulated, poorly demarcated, moderately to densely cellular, infiltrative neoplasm composed of neoplastic spindle cells that formed loose interlacing streams and bundles within a mild fibrovascular stroma. The high power view demonstrated that the spindle cells possess cigar-shaped nuclei, moderate cytologic atypia, and mitotic figures. The tumor cells had indistinct cell borders and a moderate amount of amphophilic granular cytoplasm. At the inner border of the tumor capsule, prominent heterophile infiltration was observed. Neoplasmic cells extend to the mass margins. The angiolymphatic invasion was not observed. The tumor was diagnosed as soft tissue sarcoma, and the tumor would be considered a grade-1 if using the canine grading scheme [10].

**Figure 5:**
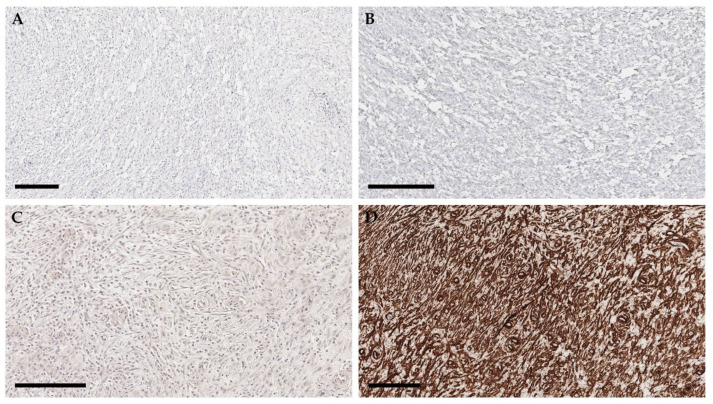
Immunohistochemistry of the mass (scale bar = 200 μm). Immunohistochemical staining of the mass. The tumor cells were negative for desmin (**A**) and vimentin (**B**), weakly positive for S100 (**C**), and robustly positive for smooth muscle actin (**D**).

For further characterization, immunohistochemistry (IHC) was performed by KVL (Figure 5). As a positive control, smooth muscle (intestine) and striated muscle tissues were used (Supplementary Figure S1). The markers used in this study were desmin (skeletal muscle marker), smooth muscle actin (smooth muscle marker), vimentin (mesenchymal cell marker), and S-100 (peripheral nerve marker). Monoclonal mouse antihuman antibodies (Agilent, Santa Clara, CA, USA), targeting each marker, were used in this study. IHC revealed that the neoplastic population exhibited diffuse, robust, cytoplasmic immunoreactivity to smooth muscle actin, patchy weak immunoreactivity to S100, and lacked immunoreactivity to desmin and vimentin (Figure 5). This immunohistochemical profile is supportive of the diagnosis of leiomyosarcoma.

## 3. Discussion

Leiomyosarcoma is a common type of malignant soft tissue sarcoma [5]. As the tumor is derived from smooth muscle, the tumor usually originates in the visceral organs or, occasionally, bones [5,6,7,8,11]. Therefore, subcutaneous leiomyosarcomas are rare but are most likely to arise in the extremities; cases occurring in the facial area are exceedingly rare [8]. In this case, we describe a facial subcutaneous leiomyosarcoma in a ball python. There were several challenges in making an accurate diagnosis. As the occurrence of facial subcutaneous leiomyosarcoma is rare, inflammation induced by trauma or infection was considered first. Furthermore, purulent materials and bacterial cells were aspirated from the mass by FNA. The bacteria were identified as *Klebsiella oxytoca*. Because this bacterium is considered an opportunistic pathogen arising from normal flora on snake skin, the mass was suspected to be an abscess [12,13]. Furthermore, after referring to human cases, we found that several reports have indicated that abscess-mimicking tumors and tumor-mimicking abscesses can cause confusion in differential diagnoses [14,15,16,17,18,19,20]. 

Histopathological examination was performed to confirm the neoplasm. Because leiomyosarcoma is a mesenchymal tumor, positive results were expected for vimentin, a mesenchymal cell marker. However, the tumor was negative for vimentin on IHC staining. Two hypotheses were considered. First, these results could indicate that the tumor originated from the muscularis propria. Council and Hameed (2009) mentioned that a lack of vimentin immunoreactivity in the muscularis propria is a differentiating characteristic to identify the origin of the tumor [21]. Therefore, although the tumor originated from smooth muscle cells, it was possible to lack reactivity to vimentin. Second, as an antihuman antibody was used in this study, the result could have been false-negative [22]. According to previous studies, the negative results could be due to the non-specificity of the antibody [22,23,24,25,26]. Moreover, the positive controls, smooth and striated muscle samples, in this case have demonstrated to be negative to vimentin, meaning non-specificity of the antibody in this reptile species (Supplementary Figure S1). Therefore, in reptile cases, it is necessary to perform and evaluate immunohistochemistry using positive controls.

Precise diagnosis is very important for appropriate treatment; depending on the diagnosis of tumor or abscess, the subsequent treatment and disease prognosis will largely differ. As the tumor growth progressed, it is likely that the central area was filled with degenerative materials, necrosis, or hemorrhage, thereby mimicking an abscess [27]. For differentiation, imaging modalities were utilized. Although it is difficult to distinguish tumor lesions from abscesses using CT imaging [27], a thorough radiographic evaluation is still recommended to evaluate the bone status and to detect metastatic lesions in lungs and other visceral organs.

## Data Availability

Not applicable.

## Supplementary Materials

The following are available online at https://www.mdpi.com/article/10.3390/vetsci8100224/s1, Figure S1. Immunohistochemistry of the control samples.
