# Supplementary material for: A Case of Submandibular Leiomyosarcoma, Mimicking an Abscess, in a Ball Python (Python regius)
Source: Vet Sci. 2021 Oct 11;8(10):224. doi: 10.3390/vetsci8100224 (PMC8539710; doi:10.3390/vetsci8100224)
Supplement: Supplementary file 1 [file vetsci-08-00224-s001.zip › vetsci-1334387-supplementary.pdf]

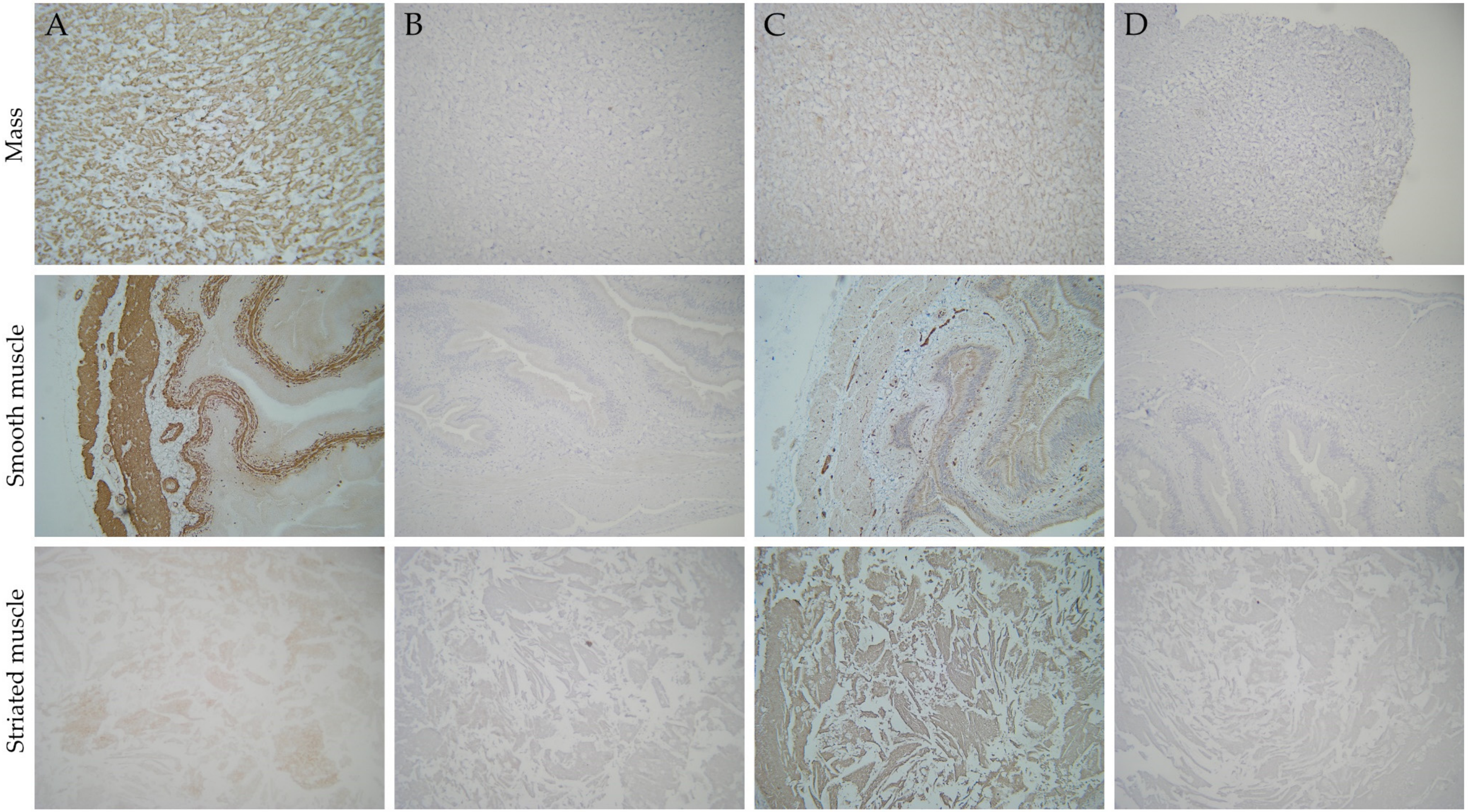

Figure S1. Immunohistochemistry of the control samples. IHC results of tumor, smooth muscle (intestine) and striated muscle. (**A**) Smooth muscle actin. Tumor and smooth muscle demonstrated positive. (**B**) Desmin. (**C**) S100. (**D**) Vimentin.
